# Acute arterial occlusion due to left ventricular thrombus of Takotsubo cardiomyopathy in a young adult: a case report

**DOI:** 10.1186/s40981-018-0190-1

**Published:** 2018-07-06

**Authors:** Yuudai Horiguchi, Takuo Hoshi, Aya Yoshimatsu, Mika Yoshida

**Affiliations:** 1 Department of Anesthesiology and Critical Care Medicine, Ibaraki Prefectural Central Hospital, 6528 Kasama, Ibaraki, 309-1793 Japan; 2 Department of Anesthesiology and Critical Care Medicine, Clinical and Educational Training Center, Tsukuba University Hospital, Tsukuba, Japan

**Keywords:** Takotsubo cardiomyopathy, Acute arterial occlusion, Left ventricular thrombus

## Abstract

**Background:**

Thromboembolism is a rare complication of Takotsubo cardiomyopathy. Importantly, an acute arterial occlusion needs rapid diagnosis and urgent treatment to help save the patient’s life. Here, we report a case of arterial occlusion due to ventricular thrombus of Takotsubo cardiomyopathy.

**Case presentation:**

A woman in her 30s, without previous medical history, felt sudden strong pain on her right leg and was diagnosed with right femoral arterial occlusion. An emergency operation was subsequently performed to take out thrombus. The patient’s oxygenation deteriorated to 93% of hemoglobin saturation just after extubation and exacerbated in the intensive care unit. Transthoracic echocardiography revealed Takotsubo cardiomyopathy-like left ventricular wall motion abnormalities and left ventricular thrombus. Heparin treatment was immediately started. After 10 days, the thrombus disappeared and the left ventricular wall motion improved and she was discharged from the hospital.

**Conclusions:**

The patient’s acute arterial occlusion in this case report was mainly caused by thrombus of cardiac origin. We suggest to routinely check echocardiography reports before surgery and perform anesthetic management carefully to better control the patient’s blood pressure and heart rhythm.

## Background

Takotsubo cardiomyopathy was first reported in Japan in 1990 and has since become increasingly recognized worldwide [1]. This cardiomyopathy is normally described as apical ballooning due to apical akinesis or hypokinesis with preserved basal segments. In addition, thrombus in the akinetic ventricular apex is observed in 2–8% of Takotsubo cardiomyopathies, but it rarely leads to the occurrence of arterial embolism [2]. On the other hand, acute arterial occlusion of extremities must be treated rapidly to save not only the affected extremities but also the patient’s life [3]. Here, we report a case of acute femoral arterial occlusion in a young woman who had a hypoxemia perioperative period that was due to left ventricular dysfunction and left ventricular thrombi in Takotsubo cardiomyopathy.

## Case presentation

**Fig. 1:**
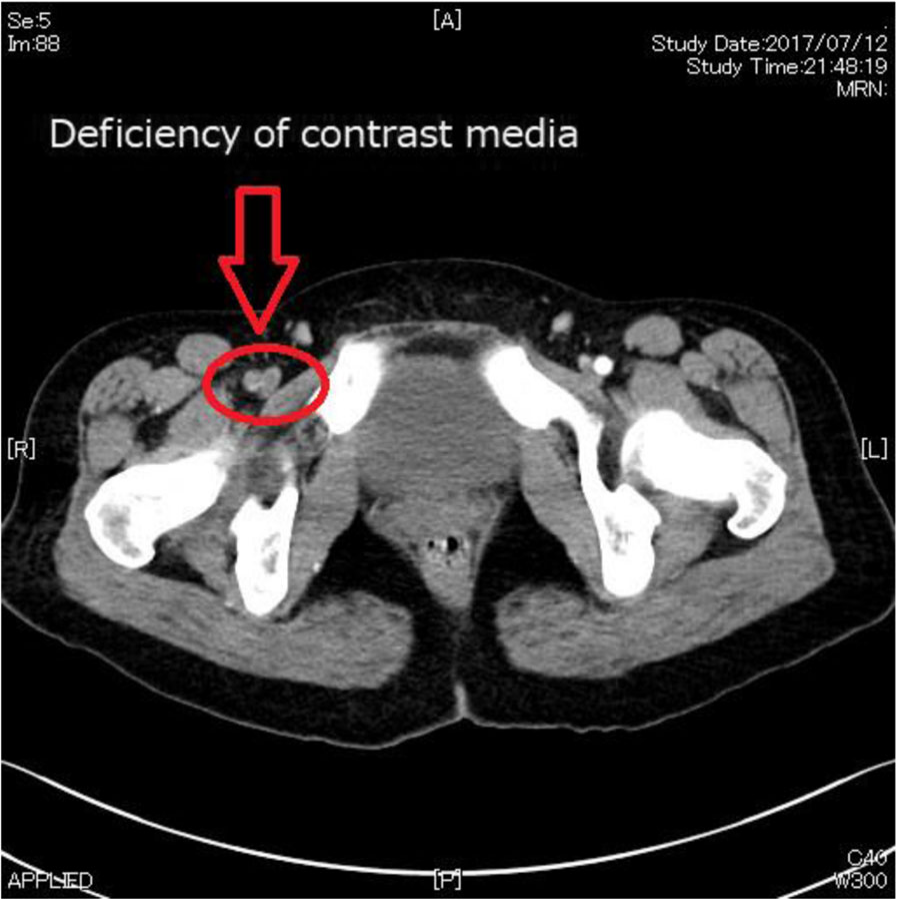
Contrast-enhanced computed tomography (CT) showing a deficiency of contrast media at the right femoral artery

**Fig. 2:**
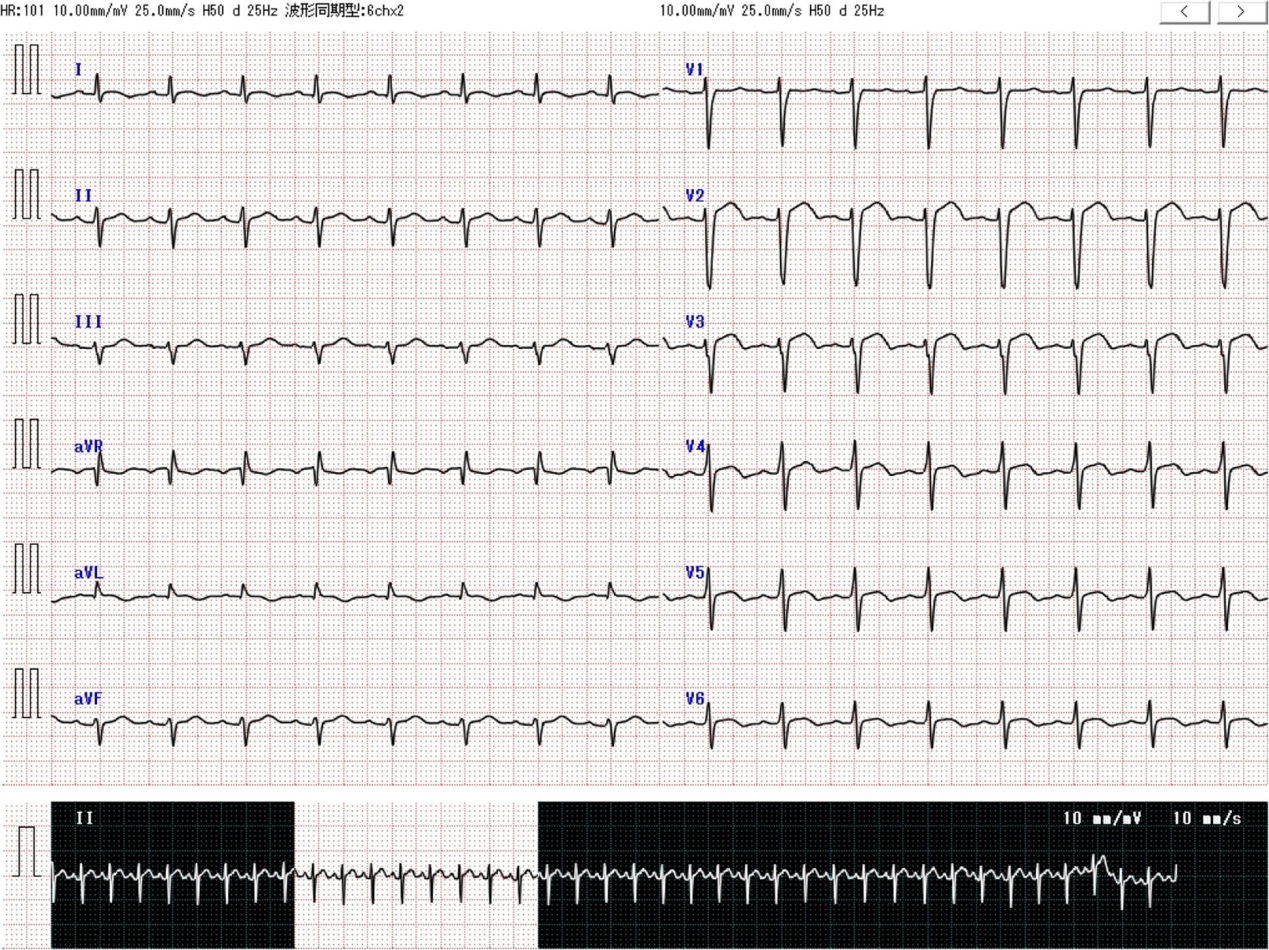
Pre-operative electrocardiogram (ECG) depicting the non-specific ST elevation and left bundle branch block observed

**Fig. 3:**
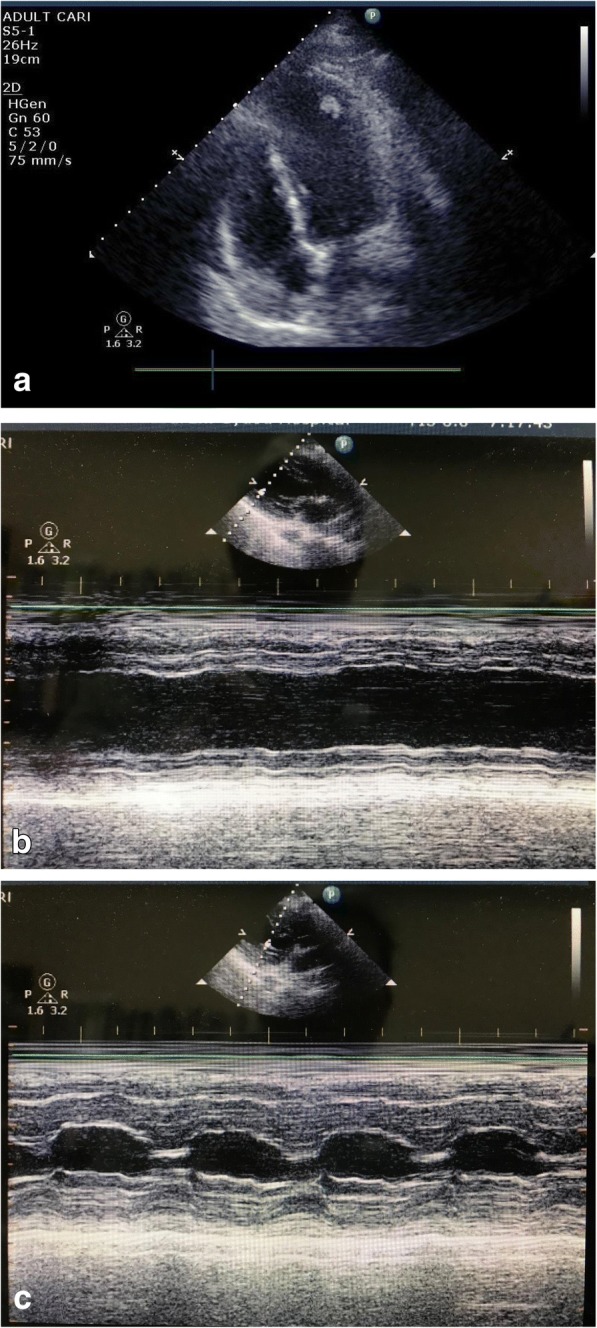
Results from the transthoracic echocardiography (TTE) performed at the ICU. A thrombus can be seen at the apex of the left ventricle. **a** Left ventricular thrombus. **b** Severe hypokinesis in apical segments. **c** Preserved contractile in basal segments

A woman in her 30s (height 163 cm, weight 64 kg) was presented as an emergency due to acute and intense right leg pain. The patient had no significant history of emotional or physical stress events 48 h before emergency visit and also no history of past personal or family history of thrombus formation (i.e., thrombogenesis), but used oral contraceptives. Her pre-operative coagulation profile revealed normal prothrombin time of 10.8 s, prothrombin time-international normalized ratio 0.93, and activated partial thromboplastin time 27.0 s, but fibrin/fibrinogen degradation products and D-dimer level were increased to 16.9 and 4.9 μg/mL. Contrast-enhanced computed tomography revealed right femoral artery thrombus (Fig. 1), and emergency thrombectomy was scheduled. Pre-operative electrocardiogram showed non-specific ST elevation and a left bundle branch block (Fig. 2), but no abnormal findings were seen on chest X-ray. On arrival in the operating room, the patient was alert and her blood pressure was 115/85 mmHg. Heart rate was 98 beats per minute, and oxygen saturation was 93% on 2 L/min oxygen by nasal cannula. The patient complained of strong right leg pain, but no dyspnea or chest pain. General anesthesia was inducted using a rapid sequence method with remifentanil, propofol, and rocuronium and maintained with remifentanil and sevoflurane as per standard protocol. After tracheal intubation, poor pulmonary oxygenation was observed, with PaO_2_87.0 mmHg and F_I_O_2_ 0.44; therefore, a recruitment maneuver was performed and oxygenation improved to PaO_2_ 174 mmHg (Table 1). During the operation, her vital signs remained stable, and she was transferred to the intensive care unit (ICU) after successful surgery. In the ICU, the patient’s oxygen saturation decreased to 90% on oxygen 5 L/min with use of a facemask. As pulmonary embolism was suspected, transthoracic echocardiography (TTE) was performed. TTE showed Takotsubo cardiomyopathy-like left ventricular dysfunction with left ventricular thrombus (Fig. 3a–c). Although she showed severe left ventricular dysfunction, creatine kinase-MB (CK-MB) and troponin T elevated modestly (creatine kinase and CK-MB were 270, and 30 U/L, troponin T was 0.23 ng/mL). Continuous intravenous heparin was immediately started at 20,000 U/day for 10 days.

**Table 1:** Gas analysis results obtained from the patient’s arterial blood at various events during her hospital stay

	After intubation (FiO_2_ 0.44)	Before extubation (FiO_2_ 0.4)	During ICU entrance (O_2_ 5 L/min; face mask)
pH	7.396	7.369	7.379
PCO_2_ (mmHg)	31.9	37.4	34.6
PO_2_ (mmHg)	87.0	174	64.4
ABE (mmol/L)	− 4.3	− 3.3	− 4.0
HCO_3_^−^ (mmol/L)	19.2	21.0	20.0
SO_2_ (%)	96.3	98.8	90.1
Lactate (mmol/L)	3.0	2.2	2.8
P/F ratio	197.7	435	161

Noninvasive positive pressure ventilation was used for 2 days until her oxygen saturation improved. Ten days after surgery, the patient was discharged from the hospital as the left ventricular thrombus disappeared and left ventricular wall motion improved. No inherited or secondary thrombophilia was identified.

## Discussion

Takotsubo cardiomyopathy is described as a transient reversible cardiomyopathy that typically occurs in older women after emotional or physical stress [4]. The wall motion abnormality of Takotsubo cardiomyopathy usually extends to the territory comprising the coronary arteries, and it is typically described as apical ballooning due to apical akinesis or hypokinesis with preserved or hypercontractile basal segments [2]. The criteria used to diagnose Takotsubo cardiomyopathy have been described in detail by the Mayo Clinic [5], and it is essential to exclude significant organic stenosis or coronary artery spasm. Although women have a 9-fold higher risk of developing this cardiomyopathy when compared to men, women who are less than 55 years of age have a 5-fold risk reduction of developing the cardiomyopathy when compared to women who are older than 55 years of age [6]. Therefore, the case reported here was not at a common age for development of Takotsubo cardiomyopathy. However, given that there are several reports of Takotsubo cardiomyopathy developing perioperatively [7, 8], anesthesiologists must be prepared to provide appropriate management such as trans esophageal echocardiography and/or regional cerebral oxygen saturation monitoring. In approximately 85% of acute arterial embolism cases, occlusion is caused by emboli from a cardiac source [9], including atrial fibrillation associated with valvular heart disease or mural thrombi in an infarcted left ventricle. Therefore, the presence of cardiac thrombus should always be assessed to appropriately evaluate the source of acute arterial occlusion. It takes 2–5 days to develop thrombi after onset of Takotsubo cardiomyopathy [2], and acute leg pain was not the cause of Takotsubo cardiomyopathy.

Since our patient had stable vital signs without the use of catecholamines and since a typical wall motion abnormality such as apical ballooning extends across the territory of three vessels, and only modest elevation in cardiac troponin was observed as acute myocardial infarction, we did not perform coronary angiography. We found the gradual recovery of ventricular function by frequent TTE. Ten days after surgery, her ventricular function returned normal and Takotsubo cardiomyopathy was diagnosed without coronary angiography.

Left ventricular thrombus formation and cardioembolic complications are atypical findings in patients presenting with Takotsubo cardiomyopathy. Gregorio et al. and Heckle et al. examined the incidence of thrombus and embolic events in patients with Takotsubo cardiomyopathy and found it to be approximately 2.5–9.2 and 0.8–5.7%, respectively [10, 11]. Importantly, patients with left ventricular thrombi have an increased embolic risk and should be treated with anticoagulation [12]. Heckle et al. also recommended the start of anticoagulation therapy for patients with this type of cardiomyopathy [11]. Given that there can be a sudden rise in blood pressure, thought to be caused by the isolation of the left ventricular thrombus [13], proper management of hemodynamics is imperative during the perioperative and recovery period in Takotsubo cardiomyopathy.

Reports suggest that approximately 71% of Takotsubo cardiomyopathy patients have significant stressful events less than 48 h before the diagnosis is made [14].

In the present case, the patient was only aware of a significant stressful event when leg pain suddenly began. Takotsubo patients may not be identified, especially in emergency situations; therefore, the medical examiner should always keep in mind the presence of Takotsubo-related myopathies.

In addition, the patient’s oxygen saturation levels deteriorated during the perioperative period. We thought her relatively low oxygen saturation would be because of pain. There were no concerns about congestive heart failure, especially since this was an emergency operation in a healthy young woman. Furthermore, the recruitment maneuver performed during surgery improved her oxygenation levels and it appeared safe to conclude that her hypoxemia was due to the atelectasis from the severe pain she was experiencing. She did not complain severe pain after surgery, but her oxygen saturation was still low. We suspected pulmonary embolism, but TTE revealed severe left ventricular dysfunction (Fig. 3b, c); therefore, we concluded the desaturation was due to left ventricular failure. Nonetheless, special care is required in a patient with acute arterial occlusion, particularly because it can originate from a cardiac source and result in cardiac failure [9].

Oral contraceptives can decrease anticoagulant activity and promote a decrease in tissue plasminogen activator inhibitor and are known to be a risk factor for venous thromboembolism [15]. Although we could not find any inherited or secondary thrombophilia in this case, oral contraceptives might have contributed to formation of embolism.

## Conclusions

Here, we report a case of acute femoral arterial occlusion in a young woman who had hypoxemia during the perioperative period, due to left ventricular dysfunction and left ventricular thrombi caused by Takotsubo cardiomyopathy. Anesthesiologists should be aware of occult Takotsubo-like cardiomyopathies in patients with acute arterial occlusions presenting a left ventricular thrombus. In addition, echocardiography should be performed before surgery and anesthetic management should be carefully monitored to better control patient’s hemodynamics.

## Abbreviations

CK-MB: Creatine kinase-MB
ICU: Intensive care unit
TTE: Transthoracic echocardiography
